# Illegal Medicine

**Published:** 1899-11

**Authors:** Nelson W. Wilson

**Affiliations:** Buffalo, N. Y.


### ILLEGAL MEDICINE.

Conducted by NELSON W. WILSON, M. D., Buffalo, N. Y.

WEST VIRGINIA and Kentucky have been having seances with illegal practitioners of medicine, and have been invariably successful in the prosecution of the cases.

In West Virginia, itinerant physicians are required to pay a license fee of $50.00. The osteopaths started in practising in that state secure in the belief that they would not be molested, after having met a series of defeats in Kentucky, which today is the most ably defended state in the Union. It is almost wholly impossible for an illegal practitioner to do business in the blue-grass state.

It remains, however, for a newspaper, the *News*, of Clarksburg, W. Va., to begin a serious crusade against osteopaths, and it starts off the fight with a severely censorious editorial, the closing sentences of which are as follows:

The laws of West Virginia require that no physician will be permitted to practise until he shall have successfully passed an examination and secured a certificate legally giving him the privilege of following his profession. These new physicians have never passed such an examination and are therefore violating the law, imposing upon the licensed physicians, and appropriating rights which the state has vested in others. If they wish to practise let them take the examination. Judge Thompson held that the word practice, relative to medicine, meant "that branch of science which relates to the prevention, cure or alleviation of diseases of the human body." As osteopathy at least assumed to alleviate and cure disease, it was held to be amenable to the statute. Axtell was fined $50 and forbidden to practise in Kentucky. The statute of this state provides that manipulation or other expedient shall constitute the act of practising medicine. The penalty for unlawfully practising medicine in this state is a misdemeanor, punishable by the imposition of a fine not less than $100 nor more than $500; imprisonment in the county jail not less than one nor more than twelve months, or both at the discretion of the court. It is, therefore, clearly shown that these osteopaths are violating the law. In justice to the profession the law should be enforced.

There are laws in this state which cover all these points, but for reasons, which are in all probability political, there is miscarriage after miscarriage of justice in cases which censors and health authorities bring before the proper tribunals.

---

### AN INDIANA OPINION.

THE newspapers of two weeks ago reported from Frankfort, Ind., the result of a faith curist case which was on trial. A man named Chenowith and his wife were arrested and placed on trial charged with failure to provide medical attendance for their child, which had died without any attention, save that slender thread of hope which faith curists and christian scientists dangle before the eyes of their victims —a flowery prayer, made up of conceit, egotism and blasphemy. The trial had been in progress a week when it was suddenly terminated by Judge Kent, at the close of the prosecution's evidence. The jury was directed to bring in a verdict of not guilty.

The court held that there is no law in Indiana requiring a parent to procure medical attention for his children.

Judge Kent announced from the bench that there never before had been a trial like this in the county.

The state will appeal to the supreme court.

### MOTHER EDDY'S WORK ANALYZED.

THE following from the *Medical Record* is an able résumé of an able article :

In the August number of the *North American Review*, Mr. W. A. Purrington continues his consideration of the case against christian science. Taking as his text the proposition that "Publicity will destroy the cult far more quickly than legislation," he proceeds to lay bear the absurdities inherent in Mrs. Eddy's teaching by quotations from her writings. The fact that after a curriculum of only three weeks and on the payment of a fee of $300, the title of doctor of science was granted at Mrs. Eddy's college to those desirous of receiving that degree and willing to part with the money for that purpose should, if nothing else will, open the eyes of the public both to the ridiculous and the nefarious aspects of the question. Undoubtedly, however, the point that especially appeals to the masses in regard to christian science is the ingenious mingling of religion with the mundane art of healing. Unfortunately religion is and always will be made the cloak for humbug of every description. The conclusions arrived at by Mr. Purrington are as follows :

1. As a mere religious or philosophic theory christian science never would have had any vogue. Its fascination lies in its pretended cures. No one suggests taking action to restrain it as a form of worship. The most that has been suggested in regard to it has been that its ignorant votaries should not be allowed to trifle with the life and health of adults, children, and the entire community by assuming the treatment of all classes of disease, including surgical cases and contagious and infectious maladies. To say that it is an infringement of religious liberty to require the same skill and knowledge of christian scientists engaging in the business of curing the sick for hire as is required by Presbyterians or Catholics in the same business, shows ignorance of the constitutional doctrine of the Mormon case, or carelessness of statement, or such willful misstatement as Mrs. Eddy personally was guilty of when she said infringement of copyright is theft, punishable criminally.

2. Christian science has no healing power peculiar to itself, as distinguished from faith cure or any other method of diverting the mind from the ills of the body. If it had such divine power, its application would be universal; it would be as effective in surgery as in physic.

3. Why is it, then, that christian science is credited with these marvelous cures, if its foremost professors cannot bring forward any better proofs of them than are afforded by certificates no better in manner and degree than those accompanying every quack nostrum that is advertised? The explanation is simple. In perhaps the majority of cases to which physicians are called nothing more is needed than regimen and the mental stimulus that comes to the patient with the knowledge that he his under skilled care. If a physician falls ill, he calls another to attend him chiefly for the sake of this mental stimulus and to eliminate the personal factor. . . . . . . . . . . . . . . . . . In all self-limited or feigned cases whatever removes the mental tension may be beneficial. Many patients would get well without any attendance at all. To take an illustration. Childbirth, in which christian scientists profess great success, is not a disease, but the operation of a normal function. In the absence of complications, attendance is not necessary, although it may be desirable. But in the presence of certain complications the very highest skill is necessary to save life. Will any sane person say that because in the vast majority of cases children may be brought into the world safely under the attendance of a christian scientist, such a person is to be pardoned who undertakes such a complication as that of placenta previa with neither medical skill nor knowledge? Will any parent be willing, in case a child's artery is severed, to call a christian scientist rather than a surgeon? Upon the answers to these questions depends the acceptance by reasoning persons of Mrs. Eddy's theory and claim that her followers can cure all forms of human maladies and injuries, and that they should be allowed to treat medical and surgical cases without the responsibility for malpractice that rests upon "medical men." *The Outlook* has these pertinent remarks in regard to the danger of calling in christian science aid to the sick: Suppose a Mormon were to set up as a pilot, claim divine guidance, and insist on his right to take steamships in and out of New York harbor on the strength of his proficiency in the Book of Mormon, would it be a violation of liberty of the individual to prohibit and put him in jail if he persisted? Yet the danger to the community from incompetent pilotage of an ocean steamer would not be so great as the peril from incompetent treatment of certain contagious diseases."

When those members of the public who are apt to believe in the efficacy of christian science treatment can be made thoroughly to understand that a course of three weeks' training is not sufficiently long to render a person, even with spiritual aid, qualified to treat injuries and diseases, and that by employing such practitioners in many instances not only are they endangering their own lives, but also those of their neighbors, perhaps they will pause and ponder.

In the meantime strong measures should be taken to prevent parents and guardians from employing unskilled advice for their children or wards.

---

### A DOCTOR WITH BELIEFS.

DR. N. C. STEELE, of Chattanooga, Tenn., writes as follows to the *Medical Record.* His letter is published in full because it is a literary curiosity and because it shows how queerly christian science affects the human mind, even when, as in this case there is reason to believe it has had some medical training :

I have just read your article on Faith Curing in Illinois. I am not a christian scientist. There may not be any basis of truth for their claims as to curing people, but I am inclined to think there is such a basis. Of course Mrs. Eddy's book is mostly a conglomeration of nonsense, but the fact that christian scientists cure the sick is what influences people to accept their doctrine or theory as true. As far as human evidence can establish a proposition their "healers" cure as large a proportion of the sick as drug physicians. Or what is the same thing to the sick as the "science," they are made to believe that they are cured.

Another point : I believe in giving sick people medicine, and I take some myself when I am sick. When I am very sick I like to take as little medicine as possible. Now suppose, although perfectly sane, I would conclude that I would not take any medicine at all. Would any person or organisation have any right to make me take it? I think not. I have never heard of such a proceeding.

If my child is sick and I think it should have medicine I effectually insist on its taking the medicine whether the child wants to take it or not. I do this because I feel responsible for the welfare of my child. I do not think the child competent to judge for itself, and so I exercise my judgment for it.

All will probably agree that I am right in this position. All will agree that I have a right to change doctors, and that I have the right to change from a "regular" to a "homeopath" or "eclectic," or the reverse. Some would question my wisdom in thus changing, but no one would think of doing so insane a thing as invoking "the law" to compel me to make a change or not make a change of said doctors. Why? Because they recognise my right to act for my child. Now suppose that for the good of the sick child I dismiss the drug-doctor and call in a faith or mind "healer," one who does not give drugs. At once some one raises the cry that I should be forced to have a "drug-doctor." Is this right? Is it just? Is it sanity? No, it is all wrong. Persuade me, if you can, to have a drug-doctor for myself or child, but don't try to force me to do either. You had better not. The argument used to justify compelling people to have a drug-doctor is that the sick person may die; yes, sir, may die! If a "mind healer" lets a patient die, a great hue and cry is raised against him, just as if no drug-doctor ever lost a case. If a drug-doctor and a "healer" should each treat 100 cases, the patients under each doctor being as nearly similar as possible, and the drug-doctor should lose ten of his hundred and the "mind-healer" should lose only one of his hundred, there would be a great outcry against the "healer" and the family of the dead one, while hardly a remark would be made about the drug-doctor losing ten cases. The verdict would probably be that he had "pretty good luck" with his 100 cases, or "he did the best he could." Multiplied thousands of people die in New York every year in spite of the best efforts of her many competent and painstaking doctors, and no one thinks anything of it, much less does any one denounce those doctors for letting those thousands die. But if a christian scientist or other "healer" lets one sick person die while being "treated" without drugs, at once every one connected with the case is denounced, and there is talk of "the law," and the incident is published far and near.

The childish absurdity of all this should be plain to every one, but it is not. We are blind worshipers of tradition, faithful trailers in the old ruts, and loyal believers in the sacred and exclusive orthodoxy of drug medication as were our fathers. History repeats itself. Our grand children will smile when they read of our absurdities, eccentricities, and contradictions in these things. If a man wants for himself or child a doctor who does not give drugs, argue with him if you wish, convince him of his folly if you can, but don't talk about forcing him with "the law."

As to the "mind-healer," "faith curist," or "christian scientist" (?) he will not "treat" any cases unless he is asked to do so, and he will not be asked to treat many unless he cures some; and the medicine doctor loses some of the cases treated.

I say all this although I am a strong believer in drugs. I take and give drugs, and if sick I would have a drug-doctor. I plead for justice, fair-dealing and common-sense in this matter.

---

### CHRISTIAN SCIENCE ORDINANCE.

THERE was another hearing before the Buffalo Board of Aldermen Committee on the christian science ordinance on October 19th. The *News* summed up the meeting as follows:

The local christian science controversy, which had its orgin somewhere in the remote past and has dragged along ever since, had another link added to its length yesterday afternoon. The aldermen of the committee on ordinances must have contracted the habit of listening to arguments pro and con in the christian science matter, for they have been hearing the same arguments, the same criminations and recriminations over and over again for months, and still, apparently, they crave more.

If the hearing differed from several previous hearings it was in respect to increased bitterness of feeling. Attorney John Cuneen called Health Commissioner Wende a "heartless, unfeeling tyrant." Rev. Dr. O. P. Gifford, pastor of the Delaware Avenue Baptist Church, mercilessly ridiculed the scientists and questioned the honesty of their gains in money, and other ministers demanded that the scientists obey the law as good citizens should.

Mr. Cuneen said that the spirit exhibited by the clergymen was the same one that had sent unfortunate men to the torture and to death and would send them there again if it could. Dr. Henry R. Hopkins in substance gave Mr. Cuneen the lie. Mr. Cuneen in substance told Dr. Hopkins that he was the reverse of a gentleman. Even the soft-voiced and gentle Attorney Tanner permitted himself to give voice to the awful charge that the Health Department has lately shown an "animus."

